# Toll-like receptor gene polymorphisms are associated with susceptibility to graves' ophthalmopathy in Taiwan males

**DOI:** 10.1186/1471-2350-11-154

**Published:** 2010-11-05

**Authors:** Wen-Ling Liao, Rong-Hsing Chen, Hui-Ju Lin, Yu-Huei Liu, Wen-Chi Chen, Yuhsin Tsai, Lei Wan, Fuu-Jen Tsai

**Affiliations:** 1 Genetic Center, China Medical University Hospital, Taichung, Taiwan; 2 School of Chinese Medicine, China Medical University, Taichung, Taiwan; 3 School of Post Baccalaureate Chinese Medicine; China Medical University, Taichung, Taiwan; 4 Department of Ophthalmology, China Medical University Hospital, Taichung, Taiwan; 5 Graduate Institute of Integrated Medicine; China Medical University, Taichung, Taiwan; 6 Department of Biotechnology, Asia University, Taichung, Taiwan

## Abstract

**Background:**

Toll-like receptors (TLRs) are a family of pattern-recognition receptors, which plays a role in eliciting innate/adaptive immune responses and developing chronic inflammation. The polymorphisms of TLRs have been associated with the risk of various autoimmune diseases, including systemic lupus erythematosus (SLE), multiple sclerosis and rheumatorid arthritis. The aim of this study was to evaluate whether TLR genes could be used as genetic markers for the development of Graves' ophthalmopathy (GO).

**Methods:**

6 TLR-4 and 2 TLR-9 gene polymorphisms in 471 GD patients (200 patients with GO and 271 patients without GO) from a Taiwan Chinese population were evaluated.

**Results:**

No statistically significant difference was observed in the genotypic and allelic frequencies of TLR-4 and TLR-9 gene polymorphisms between the GD patients with and without GO. However, sex-stratified analyses showed that the association between TLR-9 gene polymorphism and GO phenotype was more pronounced in the male patients. The odds ratios (ORs) was 2.11 (95% confidence interval [CI] = 1.14-3.91) for rs187084 AàG polymorphism and 1.97 (95% CI = 1.07-3.62) for rs352140 AàG polymorphism among the male patients. Increasing one G allele of rs287084 and one A allele of rs352140 increased the risk of GO (*p *values for trend tests were 0.0195 and 0.0345, respectively). Further, in haplotype analyses, the male patients carrying the GA haplotype had a higher risk of GO (odds ratio [OR] = 2.02, 95% confidence interval [CI] = 1.09-3.73) than those not carrying the GA haplotype.

**Conclusion:**

The present data suggest that TLR-9 gene polymorphisms were significantly associated with increased susceptibility of ophthalmopathy in male GD patients.

## Background

Graves' disease (GD) is an organ-specific autoimmune thyroid disease, one of the manifestations of which is ophthalmopathy [1]. Graves' ophthalmopathy (GO) is characterized by inflammation and fat deposition in the eye muscles and the connective tissue surrounding the eye. It is known that multiple factors contribute to the etiology and severity of GD, including the host's genetic factors as well as environmental factors [2,3]. Female sex, old age, and smoking history are common risk factors for GD [4-8]. With regard to genetic factors, the classical major histocompatibility complex class II genes and cytotoxic T cell antigen-4 genes (CTLA-4) [9-11] have been consistently reported to be associated with GD. Also, there were published studies on association of GO and genes such as CD103 [12], CTLA-4 and IL-13 [13]. However, the findings of most genes effect in GD or GO were inconsistent. Recently, toll-like receptors (TLRs) which play important roles in eliciting human innate/adaptive immune responses and developing chronic inflammation [14] are a new area of basic immunological investigation and could be associated with autoimmune thyroiditis [15].

TLRs are a family of pattern-recognition receptors and can be expressed in several types of cells and tissues such as macrophages, dendritic cells (DCs), B cells, T cells and monocytes. A total of 10 human TLRs have been identified and the functions of human TLR-1 to TLR-9 have been characterized [16,17,14,15]. TLRs are well known to recognize a variety of microbial molecules such as TLR-4 can recognize lipopolysaccharides (LPSs) in gram-negative bacteria and TLR-9 activation can be triggered by unmethylated CpG DNA of bacteria. Once TLRs are activated by microbial molecules, downstream signalling pathway via myeloid differentiation factor 88 (MyD88) and interleukin-1R (IL-1R)-associated kinase (IRAK) activates nuclear factor κB (NF-κB), leading to cytokine production, and even to apoptosis. TLRs could response to not only exogenous, microbe derived pathogen associated molecular patterns (PAMPs) but also endogenous or self ligands. Recently, the ability of TLRs to recognize host-derived danger signals, which are produced on cell damage and necrosis, was also recognized [18,19]. Moreover, studies have found that the immune complexes containing self-RNA and/or self-DNA can act as endogenous triggers for the activation of TLRs [20-22].

Recently, release of endogenous TLR ligands during inflammation and the consequent activation of TLR signaling pathways have been indicated and polymorphism of the TLR4 and TLR9 gene have been reported to be associated with many autoimmune diseases, such as systemic lupus erythematosus (SLE), atherosclerosis, asthma, type 1 diabetes, multiple sclerosis, and rheumatoid arthritis (RA) [14,23-31]. However, to the best of our knowledge, there were no reports about TLR4 and TLR9 in GO. Therefore, our aim in the present study was to investigate the potential association between GO and single-nucleotide polymorphisms (SNPs) of TLR genes--TLR-4 and TLR-9 genes--in a Chinese population in Taiwan.

## Methods

### Patients and data collection

Four hundred and seventy one GD patients at China Medical University Hospital in Taiwan were enrolled in present study. All GD patients were examined by experienced endocrinologist and identified using following criteria: having hyperthyroidism, having diffused goiter and presence at least one of thyroid-stimulating hormone (TSH) receptor antibody, diffusely increased ^131^I (iodine-131) uptake in the thyroid gland or presence of exophthalmos. The proptosis was quantified with an exophthalmometer. The data regarding age, sex, history of tobacco use, thyroid gland pathology and affected anatomic sites were extracted from the questionnaire and blood samples were collected by venipuncture for genomic DNA isolation and serological test at the enrollment in study. Informed consent was obtained from each participant before his/her inclusion in this study and the ethics committee of China Medical University Hospital gave its approval for the study.

### Genomic DNA extraction and genotyping

The genomic DNA was extracted from peripheral blood leukocytes of GD patients using the Genomic DNA kit (Qiagen) according to the manufacturer's instructions. To select the most representative single nucleotide polymorphisms (SNPs) by capturing the majority genetic variation, SNP genotype information was downloaded in December 2008 from the HapMap Han Chinese in Beijing (HCB) and Japanese in Tokyo (JPT) population. HapMap genotypes were analyzed within Haploview and Tag SNPs were selected using the Tagger function. The following criteria were used to select Tag SNPs: (1) a threshold minor allele frequency (MAF) of 0.10 (2) multimarker method with r^2 ^threshold >0.8 and logarithm of odds threshold 3.0 (3) probe or primers that pass the qualification as recommended by the manufacturer (Applied Biosystems Inc., Foster City, CA) to ensure a high genotyping success rate. The mean max r^2 ^were 0.949 and 1 for TLR4 and TLR9 SNPs, respectively. A total of six SNPs for TLR-4 and 2 SNPs for TLR-9, were selected in present study. Genotyping was achieved using an assay on-demand allelic discrimination assay and detection system according to the manufacturer's instructions (Applied Biosys tems). Briefly, polymerase chain reaction (PCR) was performed in the presence of 10 ng genomic DNA, 10 μl TaqMan master mix, and 0.125 μl of 40x assay mix. Polymerase chain reaction analysis was performed in 96-well plates on a thermal cycler (ABI 9700; Applied Biosystems). Reaction conditions were 50°C for 2 minutes and 95°C for 10 minutes, followed by 40 cycles at 95°C for 15 seconds and 60°C for 1 minute. Real-time detection of fluorescence signals was performed using the ABI Prism 7900 Real-Time PCR System. The frequency of SNPs' genotype for HCB + JPT population was extracted from PubMed [32-36].

### Statistical analysis

The difference of genotypic and allelic frequencies distributions between GD patients with and without ophthalmopathy and between GD patients and individuals information from HCB-HapMap were analyzed by the χ^2 ^test or Fisher exact test. The odds ratio (OR) was calculated from genotypic and allelic frequencies with 95% confidence interval (CI) by using unconditional logistical regression adjusting for age of diagnosis, gender and smoking history. We further stratified data into subgroups by sex and homogeneity test were performed to determine whether the association between genotype/haplotype frequencies and GO phenotype is similar in males and females two groups. If the homogeneity test was significant, the different association in males and female was indicated. Then males and females two groups should be analyzed separately. All statistical analyses were conducted using SAS statistical software, version 9.1 (SAS Institute Inc., Cary, NC). All tests were two-sided, and a *p *value less than 0.05 was used as the level of significance.

#### Power calculation

The power calculation was based on the following assumptions: the allele frequencies of the investigated SNPs within the population were based on the information from HapMap-CHB (0.422 to 0.675 for TLR9 SNPs and 0.067 to 0.633for TLR4 SNPs) and the size of present study were 271 for control group and 200 for case group. Therefore, the estimated effect size of OR, 0.578 to 0.592 for TLR9 and 1.63 to 2.25 for TLR4 of the investigated SNPs in case group can be observed using a two independent sample test with a power of 80% at two-sided type I error rate 0.05 (Additional file 1: table S1).

For the effect size of TLR on GO, no formal data are available. Therefore, we compare the observable effect sizes from our design power calculation with the effect sizes (OR = 0.55 to 0.56 for TLR9 and 1.78 to 2.85 for TLR4) from previous study in RA [37]. The magnitude of effect size in previous study in RA is bigger than our estimated effect size. Therefore, the power will be bigger than 80% if our effect size could be the same with the effect size from previous study in RA. Based upon above information, our study design could be justified.

## Results

A total of 471 GD patients comprising 200 patients with GO and 271 patients without GO were enrolled in this study. The female to male ratio was 3.75 and the mean age was 39.41 ± 12.42 years. There were statistically significant differences in the GO phenotype and smoking history between the male and female patients. The percentage of male and female GD patients with GO was 52% and 40%, respectively (*p *= 0.04). None of the frequency distribution for other anatomic sites (goiter, nodular hyperplasia, myxedema and vitiligo) was significant different between two genders. A higher percentage of male patients had smoking history than female patients (66.67% vs. 12.10%, *p *< 0.0001) (Table 1).

**Table 1:** Characters of Graves' Disease Patients between Male and Female

	Male (n = 99)	Female (n = 372)	*P*-value*
Ophthamology			
Yes	51 (51.52)	149 (40.05)	
No	48 (48.48)	223 (59.95)	0.04
Goiter			
Yes	95 (95.96)	345(92.74)	
No	4 (4.04)	27 (7.26)	0.25
Nodular hyperplasia			
Yes	8 (8.08)	39 (10.48)	
No	91 (91.92)	333 (89.52)	0.48
Myxedema			
Yes	2 (2.02)	4 (1.08)	
No	97 (97.98)	368 (98.92)	0.46
Vitiligo			
Yes	1 (1.01)	3 (0.81)	
No	98 (98.99)	369 (99.19)	0.84
Age at enrollment mean ± SD†	40.25 (10.62)	39.81 (12.57)	0.55†
Age at diagnosis mean ± SD†	35.69 (10.34)	34.57 (12.25)	0.16†
Smoking history			
Never	33 (33.33)	327 (87.90)	
Ever	66 (66.67)	45 (12.10)	<0.001

Data are no. (%)

*Chi square test.

†Mann-Whitney Wilcoxon test.

### Association between TLR gene polymorphism and disease severity

We compared the allelic and genotypic frequencies of TLR-4 and TLR-9 gene polymorphisms in the Taiwanese GD patients, with the information regarding the populations of Han Chinese in Beijing (HCB) and Japanese in Tokyo (JPT) extracted from the PubMed SNP database. The TLR-4 and TLR-9 gene polymorphisms in the Taiwanese GD patients were not statistically different from those in normal HCB and JPT. Further, we classified the GD patients on the basis of the presence of GO (Yes/No) in order to determine whether TLR-4 and TLR-9 genes were associated with the GO phenotype. The results did not indicate any significant differences in the TLR-4 and TLR-9 gene polymorphisms between the GD patients with and without GO (Additional file 2: table S2 and additional file 3: table S3). All tested SNPs were in Hardy-Weinberg equilibrium (p > 0.05).

### Sex-specific effects of TLR gene polymorphisms on susceptibility to GO

In the present study, 52% male GD patients had GO as compared to 40% female GD patients (*p *= 0.04). However, there was no sex-based difference in the genotypic distribution of TLR-4 and TLR-9 gene polymorphisms (data not shown). Therefore, we performed sex-stratified analyses to investigate the association between TLR gene polymorphisms and GO phenotype in the 2 sex-based subgroups. Statistically significant differences were observed in the allelic frequencies of 2 SNPs of the TLR-9 gene between the male patients with and without GO but not in the female patients (Table 2). The odds ratios (ORs) for rs187084 AàG polymorphism were 2.11 (95% confidence interval [CI] = 1.14-3.91) and 0.79 (95% CI = 0.57-1.08) for the male and female patients, respectively. The p value for homogeneity test was 0.01. For rs352140 AàG polymorphism, the ORs were 1.97 (95% CI = 1.07-3.62) and 0.76 (95% CI = 0.55-1.05) for the male and female patients, respectively. The *p *value for homogeneity test was 0.01. Among the male patients, increasing one G allele of rs287084 and one A allele of rs352140 increased the risk of GO (*p *values for trend tests were 0.0195 and 0.0345, respectively). Furthermore, we investigated the association between the TLR-9 haplotype and susceptibility to GO in the male and female patients. AG haplotype was significantly inversely associated with susceptibility to GO (OR = 0.49, 95% CI = 0.26-0.89), while GA haplotype was significantly associated with a high risk of GO (OR = 2.02, 95% CI = 1.09-3.73) in the male patients (Table 3). However, this sex-specific association was not found in the case of even one of the SNPs of the TLR-4 gene. Furthermore, we investigated the association between various phenotype parameter and GO among male but no significant association was found (Additional file 4: table S4).

**Table 2:** The Genotype and Allelic Frequency of TLR-9 Stratified by Sex among Graves' Disease Patients in Taiwan

		rs187084					rs352140			
	**SNP ID**	**with GO**	**w/o GO**	*P*-value*	**OR† (95% CI)**		**with GO**	**w/o GO**	*P*-value*	**OR† (95% CI)**
									
		**N (%)**	**N (%)**				**N (%)**	**N (%)**		

**Genotype**										
**Female**	**A/A**	67 (44.97)	89 (39.91)		1	**A/A**	12 (8.05)	24 (10.76)		0.57 (0.26, 1.24)
	**A/G**	71 (47.65)	108 (48.43)		0.86 (0.55, 1.34)	**A/G**	66 (44.30)	110 (49.33)		0.73 (0.47, 1.14)
	**G/G**	11 (7.38)	26 (11.66)	0.33	0.51 (0.23, 1.33)	**G/G**	71 (47.65)	89 (39.91)	0.30	1
**Male**	**A/A**	18 (35.29)	27 (56.25)		1	**A/A**	9 (17.65)	3 (6.25)		4.43 (1.04, 18.89)**
	**A/G**	25 (49.02)	18 (37.50)		2.15 (0.91, 5.09)	**A/G**	23 (45.10)	19 (39.58_		1.70 (0.72, 3.99)
	**G/G**	8 (15.69)	3 (6.25)	0.08	4.53 (1.03, 19.91)‡	**G/G**	19 (37.25)	26 (54.17)	0.11	1
		**P _Homogeneity Test_**		:0.01			**P _Homogeneity Test_**		:0.01	
**Allelic**										
**Female**	**A allele**	205 (68.79)	286 (64.13)		1	**A allele**	90 (30.20)	158 (35.43)		0.76 (0.55, 1.05)
	**G allele**	93 (31.21)	160 (35.87)	0.19	0.79 (0.57, 1.08)	**G allele**	208 (69.80)	288 (64.57)	0.14	1
**Male**	**A allele**	61 (59.80)	72 (75.00)		1	**A allele**	41 (40.20)	25 (26.04)		1.97 (1.07, 3.62)
	**G allele**	41 (40.20)	24 (25.00)	0.02	2.11 (1.14, 3.91)	**G allele**	61 (59.80)	71 (73.96)	0.03	1
		**P _Homogeneity Test_**		:0.01			**P _Homogeneity Test_**		:0.01	

*P value were determined by chi-square test; *P *values less than 0.05 were considered significant

†Adjusted for diagnosis age and smoking history in unconditional logistic regression model

‡trend test is significant (*p *values = for trend tests were 0.0195 ** trend test is significant (*p *values = for trend tests were 0.0345.

Abbreviations: CI, confidence interval; GO, Graves' ophthalmopathy; SNP, single-nucleotide polymorphism; OR, odd ratio.

**Table 3:** The Haplotype Frequency of TLR-9 Gene Stratified by Sex among Graves' Disease Patients in Taiwan

	Haplotype 1^*^					Haplotype 2^*^			
**SNP ID**	**with GO**	**w/o GO**	***P *value**†	**OR‡ (95% CI)**		**with GO**	**w/o GO**	***P *value**†	**OR‡**
								
	**N (%)**	**N (%)**				**N (%)**	**N (%)**		

**Female**					**Female**				
AG	203 (68.12)	284 (63.68)	0.21	1.27 (0.92, 1.74)	GA	88 (29.53)	156 (34.98)	0.12	0.76 (0.55, 1.04)
Non-AG	95 (31.88)	162 (36.32)		1	Non-GA	210 (70.47)	290 (65.02)		1
**Male**					**Male**				
AG	60 (58.82)	71 (73.96)	0.02	0.49 (0.26, 0.89)	GA	40 (39.22)	24 (25.00)	0.03	2.02 (1.09, 3.73)
Non AG	42 (41.18)	25 (26.05)		1	Non GA	62 (60.78)	72 (75.00)		1
	**P _Homogeneity Test_**		:0.01			**P _Homogeneity Test_**		:0.01	
**All**									
AG	128 (32.00)	180 (33.21)	0.70	0.94 (0.71, 1.25)	GA	263 (65.75)	355 (65.50)	0.94	1.02 (0.77, 1.35)
Non AG	272 (68.00)	362 (66.79)		1	Non GA	137 (34.25)	187 (34.50)		1

*Order of SNPs comprising the TLR9 haplotypes: rs187084 (A/G) and rs351401 (A/G). The haplotypes were identified by the Baysian statistical method available in the program Phase 2.1.

†The chi-square test (2×2 table) was performed to obtain the *p*-value. *P *values less than 0.05 were considered significant.

‡Adjusted for diagnosis age and smoke history (ever vs. never) in unconditional logistic regression model

Abbreviations: CI, confidence interval; GO, Graves' ophthalmopathy; SNP, single-nucleotide polymorphism; OR, odd ratio.

## Discussion

In this study, we investigated the effect of TLR-4 and TLR-9 gene polymorphisms on the organ-specific autoimmune disease GD in a Taiwanese population. TLR-9 polymorphisms and haplotype were significant associated with susceptibility to GO in males. However, none of the TLR-4 and TLR-9 polymorphism or haplotypes was associated with overall GO risk. The TLR4 is known to activate the NF-κB and subsequent gene expression such as cytokines and adhesion molecules. A TLR-4 polymorphism that prevents ligands binding and subsequent cellular signaling would result in lower NF-κB activation and subsequent NF-κB-dependent proinflammatory gene expression. However, we did not find any association between TLR4 polymorphism and GO in present study. Low statistical power may be the reason for this non significant results in TLR4 association in GO. For TLR9, our results were consistent with those of the previous studies with regard to the association between TLR9 gene polymorphisms and systemic autoimmune diseases, such as SLE in Japan [30,28] and China [38,36]. From SLE model, Tao et al study [30,28] found diminished expression of TLR9 could increase anti-dsDNA antibody in SLE mice model which may increase the risk of developing autoimmune disease. Also, it has been hypothesized that nucleic acid from dying cells may act as ligands for TLR9 to trigger IFN-1 production in SLE [39,37]. In this study, 2 TLR-9 SNPs, one (-1486TàC, rs187084) is in the promoter region and the other (1635AàG, rs352140) is in exon 2. The variation of TLR-9 gene in those region may down regulate TLR9 expression and involved in production of autoantibodies or IFN-1 which may increase the risk of GO. Therefore, future studies are required to investigate if variation of TLR9 gene, especially in exon region may affect TLR9 protein expression or function which increase the risk of GO.

TLR-9 polymorphisms were associated with increase the risk of GO in males in present study. In general, GD is more common among women; however, male sex also faces the risk of developing progressive and severe thyroid-associated orbitopathy [4,7,4,7] or responds poorly to treatment [40,38]. A previous study has reported a female to male ratio of 9.3 for patients with mild GO and of 3.2 for those with moderate GO, with the ratio being 1.4 for patients with severe GO [4,4]. Our data suggest that the role of TLR-9, which plays an important role in conferring innate immunity, would be more critical in men with GO. The susceptibility factor seems to play a different role depending on sex, or the effect could be more apparent in men and not in women because of the difference in the basic immune responses between the 2 sexes [41,39]. Further studies are required to investigate that if the TLR-9 activity and its potential relevance cytokines being more prone to male with GO.

As the smoking is potential confounding factor for susceptibility of GO, we adjusted the variable of smoking history in the multivariate model to eliminate the smoking effect on susceptibility of GO. However, the significant association between TLR-9 gene polymorphism and susceptibility to GO in the male patients was no changed. Furthermore, the strength association between TLR-9 polymorphism and GO in male was modest in present study. Therefore, a larger sample sized, especially in male population will be needed in the future study to confirm the importance of these polymorphisms as genetic markers of GD in Taiwanese population. Furthermore, the interpretation of our study results is limited because the patients were recruited from Taiwan only. Studies in ethnically disparate populations are needed before firm conclusions can be drawn.

## Conclusion

In conclusion, our findings suggested that TLR-9 gene polymorphisms were significantly associated with susceptibility to GO in the male GD patients in Taiwan.

## Competing interests

The authors declare that they have no competing interests.

## Authors' contributions

WLL carried out statistical analyses, results interpretation and drafted the manuscript. RHC, HJL and WCC recruited and maintained the clinical information of participants. YHL and YHT supervised genotyping experiments. LW and FJT carried out study design and coordination and have given final approval of the version to be published. All authors read and approved the final manuscript.

## Pre-publication history

The pre-publication history for this paper can be accessed here:

http://www.biomedcentral.com/1471-2350/11/154/prepub

## Supplementary Material

Additional file 1 **Table S1: Power calculation using GPower 3.1 software**. Power calculation using GPower 3.1 software. Click here for file

Additional file 2 **Table S2: Genotype Frequency of TLR-4 and TLR-9 Markers for Graves' Disease Patients in Taiwan**. The results of comparisons of TLR-4 and TLR-9 genotype frequency between the Graves' Disease patients with and without Ophthalmopathy. Click here for file

Additional file 3 **Table S3: Allelic Frequency of TLR-4 and TLR-9 Genes for Graves' Disease Patients in Taiwan**. The results of comparisons of TLR-4 and TLR-9 allelic frequency between the Graves' Disease patients with and without Ophthalmopathy. Click here for file

Additional file 4 **Table S4: Characters of Male Graves' Disease Patients with Ophthalmopathy and without Ophthalmopathy**. Characters of Male Graves' Disease Patients with and without Ophthalmopathy. Click here for file
