# Supplementary material for: Toll-like receptor gene polymorphisms are associated with susceptibility to graves' ophthalmopathy in Taiwan males
Source: BMC Med Genet. 2010 Nov 5;11:154. doi: 10.1186/1471-2350-11-154 (PMC2992489; doi:10.1186/1471-2350-11-154)
Supplement: Additional file 1 — Table S1: Power calculation using GPower 3.1 software. Power calculation using GPower 3.1 software. [file 1471-2350-11-154-S1.DOC]

**Table S1. Power calculation using GPower 3.1 software**

|  |  | **Estimated effect size*** | | |
| --- | --- | --- | --- | --- |
| **SNP ID** | **Allele frequency in control group from PubMed CHB population** | **Allele frequency in case group** | **Allele difference in two groups** | **OR** |
| **TLR9, rs187084** | 0.422 | 0.297 | 0.125 | 0.578 |
| **TLR9, rs352140** | 0.578 | 0.448 | 0.130 | 0.592 |
| **TLR4, r10116253** | 0.367 | 0.490 | 0.123 | 1.63 |
| **TLR4, rs1554973** | 0.078 | 0.154 | 0.076 | 2.15 |
| **TLR4, rs1927907** | 0.200 | 0.304 | 0.104 | 1.75 |
| **TLR4, rs1927911** | 0.633 | 0.744 | 0.111 | 1.69 |
| **TLR4, rs1927914** | 0.367 | 0.487 | 0.12 | 1.63 |
| **TLR4, rs7044464** | 0.067 | 0.139 | 0.072 | 2.25 |

*****Estimated effect size was calculation based on the allele frequencies from PubMed and present study size (271 for control group and 200 for case group) using a two independent sample test with a power of 80% at two-sided type I error rate 0.05.

Abbreviations: TLR, toll-like receptor; HCB, Han Chinese in Beijing; OR, odd ratio.
