# Supplementary material for: Toll-like receptor gene polymorphisms are associated with susceptibility to graves' ophthalmopathy in Taiwan males
Source: BMC Med Genet. 2010 Nov 5;11:154. doi: 10.1186/1471-2350-11-154 (PMC2992489; doi:10.1186/1471-2350-11-154)
Supplement: Additional file 2 — Table S2: Genotype Frequency of TLR-4 and TLR-9 Markers for Graves' Disease Patients in Taiwan. The results of comparisons of TLR-4 and TLR-9 genotype frequency between the Graves' Disease patients with and without Ophthalmopathy. [file 1471-2350-11-154-S2.DOC]

**Table S2. Genotype Frequency of TLR-4 and TLR-9 Markers for Graves’ Disease Patients in Taiwan**

| **SNP ID** | **with GO**  **(n = 200)** | **without GO**  **(n = 271)** | ***HCB*** | ***P*** ^*^ | ***P*** ^†^ | **OR‡ (95% CI)** |
| --- | --- | --- | --- | --- | --- | --- |
| **TLR4** |  |  |  |  |  |  |
| **r10116253** |  |  |  |  |  |  |
| A/A | 75 (37.50) | 102 (37.64) | 15 (33.33) |  |  | 1 |
| A/G | 91 (45.50) | 121 (44.65) | 27 (60.00) |  |  | 1.11 (0.73, 1.67) |
| G/G | 34 (17.00) | 48 (17.71) | 3 (6.67) | 0.97 | 0.08 | 0.90 (0.52, 1.54) |
| **rs1554973** |  |  |  |  |  |  |
| A/A | 132 (66) | 177 (65.31) | 64 (76.19) |  |  | 1 |
| A/G | 62 (31) | 88 (32.47) | 20 (23.81) |  |  | 0.96 (0.64, 1.43) |
| G/G | 6 (3) | 6 (2.21) | 0 | 0.83 | 0.30 | 1.56 (0.48, 5.02) |
| **rs1927907** |  |  |  |  |  |  |
| A/A | 6 (3) | 23 (8.49) | 1 (2.22) |  |  | 0.29 (0.11, 0.73) |
| A/G | 67 (33.50) | 82 (30.26) | 16 (35.56) |  |  | 1.08 (0.72, 1.62) |
| G/G | 127 (63.50) | 166 (61.25) | 28 (62.22) | 0.05 | 0.52 | 1 |
| **rs1927911** |  |  |  |  |  |  |
| A/A | 33 (16.50) | 48 (17.71) | 13 (15.48) |  |  | 1 |
| A/G | 92 (46) | 122 (45.02) | 41 (48.81) |  |  | 1.27 (0.75, 2.17) |
| G/G | 75 (37.50) | 101 (37.27) | 30 (35.71) | 0.94 | 0.84 | 1.17 (0.69, 2.02) |
| **rs1927914** |  |  |  |  |  |  |
| A/A | 75 (37.50) | 102 (37.64) | 30 (35.71) |  |  | 1 |
| A/G | 91 (45.50) | 121 (44.65) | 41 (48.81) |  |  | 1.11 (0.73, 1.67) |
| G/G | 34 (17.70) | 48 (17.71) | 13 (15.48) | 0.97 | 0.80 | 0.90 (0.52, 1.54) |
| **rs7044464** |  |  |  |  |  |  |
| A/A | 2 (1) | 2 (0.74) | 0 (0) |  |  | 1.40 (0.19, 10.36) |
| A/T | 52 (26) | 57 (21.03) | 6 (13.33) |  |  | 1.42 (0.91, 2.22) |
| T/T | 146 (73) | 212 (78.23) | 39 (86.67) | 0.41 | 0.21 | 1 |

**Table S2 (cont.). Genotype Frequency of TLR-4 and TLR-9 Markers for Graves’ Disease Patients in Taiwan**

| **SNP ID** | **with GO**  **(n = 200)** | **without GO**  **(n = 271)** | ***HCB*** | ***P*** ^*^ | ***P*** ^†^ | **OR‡ (95% CI)** |
| --- | --- | --- | --- | --- | --- | --- |
| **TLR9** |  |  |  |  |  |  |
| **rs187084** |  |  |  |  |  |  |
| A/A | 85 (42.50) | 116 (42.80) | 27 (32.14) |  |  | 1 |
| A/G | 96 (48.00) | 126 (46.49) | 45 (53.57) |  |  | 1.06 (0.71, 1.57) |
| G/G | 19 (9.50) | 29 (10.70) | 12 (14.29) | 0.90 | 0.16 | 0.88 (0.45, 1.69) |
| **rs352140** |  |  |  |  |  |  |
| A/A | 21 (10.50) | 27 (9.96) | 11 (13.10) |  |  | 0.95 (0.50, 1.81) |
| A/G | 89 (44.50) | 129 (47.60) | 45 (53.57) |  |  | 0.89 (0.60, 1.31) |
| G/G | 90 (45.00) | 115 (42.44) | 28 (33.33) | 0.80 | 0.21 | 1 |
| Data are no. (%)  ^*^ Compared Graves’ diseases patients with and without GO. Chi-square test.  †Compared Graves’ disease patients with HCB normal population. Chi-square test.  ‡Compared Graves’ diseases patients with and without GO. Adjusting for age of diagnosis, gender and smoking history in unconditional logistic regression model  *P* values less than 0.05 were considered significant. | | | | | | |

Abbreviations: CI, confidence interval; GO, Graves’ ophthalmopathy; HCB, Han Chinese in Beijing; OR, odd ratio; SNP, single-nucleotide polymorphism.
