# Supplementary material for: Toll-like receptor gene polymorphisms are associated with susceptibility to graves' ophthalmopathy in Taiwan males
Source: BMC Med Genet. 2010 Nov 5;11:154. doi: 10.1186/1471-2350-11-154 (PMC2992489; doi:10.1186/1471-2350-11-154)
Supplement: Additional file 3 — Table S3: Allelic Frequency of TLR-4 and TLR-9 Genes for Graves' Disease Patients in Taiwan. The results of comparisons of TLR-4 and TLR-9 allelic frequency between the Graves' Disease patients with and without Ophthalmopathy. [file 1471-2350-11-154-S3.DOC]

**Table S3. Allelic Frequency of TLR-4 and TLR-9 Genes for Graves’ Disease Patients in Taiwan**

| **SNP ID** | **with GO**  **(n = 200)** | **without GO**  **(n = 271)** | | ***HCB*** | | ***P-value*** ^*^ | | | ***P-value*** ^†^ | **OR‡ (95% CI)** |
| --- | --- | --- | --- | --- | --- | --- | --- | --- | --- | --- |
| **TLR4** |  | |  | |  | |  |  | |  |
| **rs10116253** |  | |  | |  | |  |  | |  |
| A allele | 241 (60.25) | | 325 (59.96) | | 57 (63.33) | |  |  | | 1 |
| G allele | 159 (39.75) | | 217 (40.04) | | 33 (36.67) | | 0.93 | 0.55 | | 0.90 (0.74, 1.27) |
| **rs1554973** |  | |  | |  | |  |  | |  |
| A allele | 324 (81.41) | | 442 (81.55) | | 148 (88.10) | |  |  | | 1 |
| G allele | 74 (18.59) | | 100 (18.45) | | 20 (11.90) | | 0.83 | 0.04 | | 1.03 (0.74, 1.45) |
| **rs1927907** |  | |  | |  | |  |  | |  |
| A allele | 79 (19.75) | | 128 (23.62) | | 18 (20.00) | |  |  | | 0.76 (0.55, 1.05) |
| G allele | 321 (80.25) | | 414 (76.38) | | 72 (80.00) | | 0.16 | 0.66 | | 1 |
| **rs1927911** |  | |  | |  | |  |  | |  |
| A allele | 158 (39.5) | | 218 (40.22) | | 67 (39.88) | |  |  | | 1 |
| G allele | 242 (60.5) | | 324 (59.78) | | 101 (60.12) | | 0.82 | 0.99 | | 1.05 (0.80, 1.38) |
| **rs1927914** |  | |  | |  | |  |  | |  |
| A allele | 241 (60.25) | | 325 (59.96) | | 101 (60.11) | |  |  | | 1 |
| G allele | 159 (39.75) | | 217 (40.04) | | 67 (39.88) | | 0.93 | 0.99 | | 0.97 (0.74, 1.27) |
| **rs7044464** |  | |  | |  | |  |  | |  |
| A allele | 56 (14) | | 61 (11.25) | | 6 (6.67) | |  |  | | 1.35 (0.91, 2.01) |
| T allele | 344 (86) | | 481 (88.75) | | 84 (93.33) | | 0.21 | 0.11 | | 1 |
| **TLR9** |  | |  | |  | |  |  | |  |
| **rs187084** |  | |  | |  | |  |  | |  |
| A allele | 266 (66.50) | | 358 (66.05) | | 99 (58.93) | |  |  | | 1 |
| G allele | 134 (33.50) | | 184 (33.95) | | 69 (41.07) | | 0.89 | 0.07 | | 0.98 (0.74, 1.30) |
| **rs352140** |  | |  | |  | |  |  | |  |
| A allele | 131 (32.75) | | 183 (33.76) | | 67 (40.12) | |  |  | | 0.94 (0.71, 1.25) |
| G allele | 269 (67.25) | | 359 (66.24) | | 100 (59.88) | | 0.74 | 0.09 | | 1 |
| Data are no. (%)  ^*^ Compared Graves’ diseases patients with and without GO. Chi-square test.  †Compared Graves’ disease patients with HCB normal population. Chi-square test.  ‡Compared Graves’ diseases patients with and without GO. Adjusting for age of diagnosis, gender and smoking history in unconditional logistic regression model  *P* values less than 0.05 were considered significant. | | | | | | | | | | |

Abbreviations: CI, confidence interval; GO, Graves’ ophthalmopathy; HCB, Han Chinese in Beijing; OR, odd ratio; SNP, single-nucleotide polymorphism
