# Supplementary material for: Toll-like receptor gene polymorphisms are associated with susceptibility to graves' ophthalmopathy in Taiwan males
Source: BMC Med Genet. 2010 Nov 5;11:154. doi: 10.1186/1471-2350-11-154 (PMC2992489; doi:10.1186/1471-2350-11-154)
Supplement: Additional file 4 — Table S4: Characters of Male Graves' Disease Patients with Ophthalmopathy and without Ophthalmopathy. Characters of Male Graves' Disease Patients with and without Ophthalmopathy. [file 1471-2350-11-154-S4.DOC]

**Table S4. Characters of Male Graves’ Disease Patients with Ophthalmopathy and without Ophthalmopathy.**

|  | **NonGO**  **(n = 48)** | **GO**  **(n = 51)** | ***P*-value^*^** |
| --- | --- | --- | --- |
| Goiter |  |  |  |
| Yes | 46 (95.83) | 49 (96.08) |  |
| No | 2 (4.17) | 2 (3.92) | 1.00^§^ |
| Nodular hyperplasia |  |  |  |
| Yes | 4 (8.33) | 4 (7.84) |  |
| No | 44 (91.67) | 47 (92.16) | 1.00^§^ |
| Myxedema |  |  |  |
| Yes | 0 (0) | 2 (3.92) |  |
| No | 48 (100) | 49 (96.08) | 0.50^§^ |
| Vitiligo |  |  |  |
| Yes | 0 (0) | 1 (1.96) |  |
| No | 48 (100) | 50 (98.04) | 1.00^§^ |
| Age at enrollment  mean ± SD | 40.20 (10.63) | 40.30 (10.73) | 0.89† |
| Age at diagnosis  mean ± SD | 36.61 (10.25) | 34.82 (10.46) | 0.45† |
| Smoking history |  |  |  |
| Never | 15 (31.25) | 18 (35.29) |  |
| Ever | 33 (68.75) | 33 (64.71) | 0.67^*^ |

Data are no. (%)

^*^ Chi square test. †Mann–Whitney Wilcoxon test. ^§^Fisher exact test
